# Patient journey mapping to investigate quality and cultural safety in burn care for Aboriginal and Torres Strait Islander children and families – development, application and implications

**DOI:** 10.1186/s12913-022-08754-0

**Published:** 2022-11-28

**Authors:** Sarah Fraser, Tamara Mackean, Julian Grant, Kate Hunter, Courtney Ryder, Janet Kelly, Andrew J. A. Holland, Bronwyn Griffin, Kathleen Clapham, Warwick J. Teague, Anne Darton, Rebecca Q. Ivers

**Affiliations:** 1 School of Population Health, Faculty of Medicine, UNSW, Sydney, Australia; 2 College of Medicine and Public Health, Flinders University, Adelaide, Australia; 3 Charles Sturt University, Bathurst, Australia; 4 The George Institute for Global Health, UNSW, Sydney, Australia; 5 University of Adelaide, Adelaide, Australia; 6 The University of Sydney, The Children’s Hospital at Westmead Clinical School, Sydney, Australia; 7 Menzies Health Institute Queensland, Griffith University, Brisbane, Australia; 8 University of Wollongong, Wollongong, Australia; 9 University of Melbourne, Melbourne, Australia; 10 Agency for Clinical Innovation, St Leonards, Willoughby, Australia

**Keywords:** Patient journey mapping, Aboriginal and Torres Strait Islander, Indigenous, Burn care, Quality, Cultural safety, Disparities

## Abstract

**Background:**

Quality and safety in Australian healthcare is inequitably distributed, highlighted by gaps in the provision of quality care for Aboriginal and Torres Strait Islander children. Burns have potential for long-term adverse outcomes, and quality care, including culturally safe care, is critical to recovery. This study aimed to develop and apply an Aboriginal Patient Journey Mapping (APJM) tool to investigate the quality of healthcare systems for burn care with Aboriginal and Torres Strait Islander children.

**Study design:**

Interface research methodology, using biomedical and cultural evidence, informed the modification of an existing APJM tool. The tool was then applied to the journey of one family accessing a paediatric tertiary burn care site. Data were collected through yarning with the family, case note review and clinician interviews. Data were analysed using Emden’s core story and thematic analysis methods. Reflexivity informed consideration of the implications of the APJM tool, including its effectiveness and efficiency in eliciting information about quality and cultural safety.

**Results:**

Through application of a modified APJM tool, gaps in quality care for Aboriginal and Torres Strait Islander children and families were identified at the individual, service and system levels. Engagement in innovative methodology incorporating more than biomedical standards of care, uncovered critical information about the experiences of culturally safe care in complex patient journeys.

**Conclusion:**

Based on our application of the tool, APJM can identify and evaluate specific aspects of culturally safe care as experienced by Aboriginal and Torres Strait Islander peoples and be used for quality improvement.

**Supplementary Information:**

The online version contains supplementary material available at 10.1186/s12913-022-08754-0.

## Background

Significant focus and effort are being directed towards ensuring quality healthcare worldwide [1]. In Australia quality standards and accreditation measures [2] influence care provision and regulate healthcare efficiency and effectiveness. Engagement in quality improvement (QI) supports healthcare services and providers to constructively critique the healthcare they provide and implement improvement activities [1]. Often, improvement is focused on performance and limitations of healthcare services, with process mapping and clinical redesign as QI methods [3, 4]. These methods often lack inclusion of patient experiences, with efficiency processes not always enhancing patient experiences or improving health outcomes.

In Australia, quality healthcare for Aboriginal and Torres Strait Islander peoples has increasingly been linked to cultural safety [5] and competency of healthcare services [6, 7]. The revised National Safety and Quality Health Service Standards (2^nd^ edn) [8] for health services now include six actions specific to the health of Aboriginal and Torres Strait Islander peoples’ and the cultural competency of tertiary healthcare services. This is the result of an increasing recognition that quality healthcare must consider both cultural [9, 10] and clinical safety, ensuring that all needs of an individual and family are met. This concept is supported by the Australian Safety and Quality Framework for Health Care [11] which positions consumer-centeredness as one of three key indicators of quality.

Many Aboriginal and Torres Strait Islander peoples have a holistic model of health and healing that is not fully responded to or always understood by Australia’s dominant biomedical health system [10]. The multi-dimensional holistic model includes considerations of physical, psychological, social health and wellbeing, spirituality, and cultural integrity aspects [12]. When cultural and spiritual aspects of health and healing are considered in the context of healthcare, health outcomes for Aboriginal and Torres Strait Islander peoples are improved, and an experience of culturally safe care can be achieved [5]. The theory of cultural safety, originally developed in New Zealand [13], has since been applied to healthcare in Australia [5, 9]. The principles of culturally responsive and respectful care have been adapted further, and have been used both in assessing quality in standards [14] and in Australian healthcare policy [15]. The cultural safety principles (reflexivity, dialogue, power imbalances, decolonisation and regardful care) are developed from First Nations knowledges and generations of lived experience [16].The principle of decolonisation sets cultural safety apart from other cultural frameworks as it is focussed on peoples who have been impacted by colonisation [16], and is different from cultural competency which takes an individualised perspective, and focusses on minority populations more broadly [6]. While many providers in the Australian mainstream healthcare system endeavour to provide culturally competent healthcare [6], it is unclear as to whether this leads to an experience of culturally safe care for consumers.

Reliably accessible patient-centred healthcare is a health inequity Aboriginal and Torres Strait Islander peoples face [17, 18], suggesting racism (in all forms), or at the very least, deficits in quality. Racism can be systemic, interpersonal or internalised [19]. Aboriginal and Torres Strait Islander peoples experiences of being judged, misunderstood, and stereotyped by healthcare providers in Australia’s mainstream healthcare system causes distress and disengagement of both individuals and families [20]. Further, communication breakdown in healthcare environments results in difficulty assessing symptoms, eliciting signs, reaching accurate diagnoses and providing effective care [21]. Institutional racism in health systems creates structural barriers and impacts at a population level. Together, these quality deficits contribute to inequitable health outcomes for Aboriginal and Torres Strait Islander peoples.

Aboriginal and Torres Strait Islander children experience burn injury at disproportionally higher rates than non-Indigenous children, and have in longer lengths of inpatient care [22]. Such disparities are one example of the many multifaceted and complex disparities experienced by Aboriginal and Torres Strait Islander families more broadly. Further, these disparities are intergenerational and need to be understood in the context of the family and community. So, while accreditation processes seek to ensure quality healthcare in tertiary settings [2], including those with specific cultural competency [8] and burn care [23] components, a single tool that specifically assesses both the clinical and cultural quality of burn care for Aboriginal and Torres Strait Islander children and families is lacking.

Patient Journey Mapping (PJM) is a quality assessment method used to better understand and provide a detailed account of patient healthcare journeys [24]. PJM has previously described the journey stages in which the healthcare system fails or succeeds to provide quality and responsive care to patients, and is therefore a useful method to appraise and guide organisations’ approaches to care [3, 4]. It highlights barriers and enablers to care from the perspective of both the recipient and provider of healthcare [3] and enables comparisons of critical points in time with existing best practice models and guidelines. Aboriginal PJM (APJM) can provide mechanisms for identifying gaps and facilitating improvements in Aboriginal patient journeys by depicting the complexities inherent in healthcare, with a focus on QI [25]. APJM may therefore provide an opportunity to investigate the lived experience of families of children with a burn injury within and across the health system and enable a proper exploration of disparities that are not simple in nature. This paper aims to examine the critical components relevant to, and modification of an existing APJM tool [26], specifically for Aboriginal and Torres Strait Islander children to assess quality and cultural safety in the burn care journey of Aboriginal and Torres Strait Islander families for the purpose of QI.

## Methods

### Ethical considerations

Ethics approval for this study was received from the: Aboriginal Health Research Ethics Committee; Women’s & Children’s Health Network Human Research Ethics Committee; Flinders University SBREC; Central Australian Human Research Ethics Committee; Human Research Ethics Committee of Northern Territory; Department of Health and Menzies School of Health; and the Department of Health Human Research Ethics Committee. All participants received a participant information sheet and provided written informed consent.

The research was guided by the National Health and Medical Research Council’s values and ethics for doing research with Aboriginal communities. These strategies were upheld throughout the research. The primary researcher (SF) worked in partnership with Waljen woman, mentor, PhD supervisor and author (TM) to design and undertake the project. Furthermore, this research forms part of a larger study [27] which is guided by an Aboriginal and Torres Strait Islander reference group.

### Theoretical framework

Cultural safety and knowledge interface research informs the methodological constructs underpinning this research and provides a space to bring together the health and healing constructs of Aboriginal and Torres Strait Islander peoples and key indicators for western biomedical burn care quality [28–30]. This is a space without notions of dominance or superiority, within which mutual respect, shared benefits, human dignity and discovery provide an opportunity for new and relative knowledge production [28, 29, 31]. This research engages a qualitative study design [32] with Indigenous ways of knowing, being and doing [33] in the context of evaluating the quality and cultural safety of healthcare systems and services for burn care with Aboriginal and Torres Strait Islander children and families using APJM [25, 26].

### Modifying the APJM Tool

To modify the APJM tool, we looked at the documented biomedical evidence informing burn care currently in Australia and found that acute burns, including those involving joints or young children, typically require specialist tertiary healthcare [34]. In Australia, these specialist tertiary services are metropolitan. We also found that burn care is best delivered by multi-disciplinary teams [35] comprising many healthcare professionals, who each bring a unique skill set, focus and contribution to care. Key professions in these teams include: nursing, occupational therapy, physiotherapy, medical, dietetics, psychology, and social work [35–38]. We also found that burn care can be separated into distinct critical points in time from a biomedical perspective, evident in the existing models that guide burn care [36] and research evidence [34]. These critical points, whilst referred to differently, include: the injury; emergency care; ambulatory care; admission; in-patient care; discharge; and rehabilitation. We found the evidence base for the models of burn care used in Australia [36–40] varied, however commonalities in treatment existed across the models. All evidence was incorporated into the APJM tool, and to consolidate and gain consensus on inclusion, input from clinicians and policy makers was sought through roundtables and review of the tool.

Consideration of the health and healing constructs of Aboriginal and Torres Strait Islander peoples for inclusion in the APJM tool was through use of the theoretical constructs [13, 29, 41] and engagement of the Aboriginal co-researchers who contributed to knowledge of how these could be applied. We found it was important for holistic philosophies and Indigenous knowledges [41] to be incorporated throughout the APJM tool. We also determined that the critical time points of burn care for families were less rigid points than those associated with biomedical burns care and were reflective of family needs.

The biomedical and cultural evidence was brought together at the knowledge interface, in order to take account different influences at different levels in complex health systems, all of which are interrelated and not independent of one another [29] and are especially important given factors informing burn care in Australia [Fraser]. We found that by developing the APJM tool at the knowledge interface, we were able to bring together, the experience of the recipient of care, the perspective of healthcare service, and the influence of the healthcare system in a manner which encompassed mutual respect, shared benefit, discovery, and dignity [29, 30, 41].

The compilation of this information into a single APJM tool was facilitated by use of a single spreadsheet file with two components: one to assess the provision of quality care compared with the biomedical evidence (Supplementary Material 1); and a second to record the burn care journey in-line with Aboriginal and Torres Strait Islander constructs of health and healing (Supplementary Material 2). A roundtable of researchers, clinicians and Aboriginal healthcare professionals critically refined the tool after which endorsement for application of the tool was sought.

### Application of the APJM tool

The mapping process, through application of the APJM tool to investigate the quality of healthcare systems and services for burn care with Aboriginal and Torres Strait Islander families included two phases: recruitment, and data collection and analysis.

#### Recruitment

An outer metropolitan family was recruited to participate in the application of the APJM tool. The participant, an Aboriginal child whose family (*n* = 6) accessed tertiary burn care, was chosen from an overarching study investigating burn injury in Aboriginal and Torres Strait Islander children [27]. The role of the main researcher in applying the tool (author SF), was a PhD candidate, external to the tertiary healthcare site, yet linked to the site through their participation in the overarching study [27]. Clinicians identified through case note review and involved in the provision of care were also invited to participate.

#### Data collection and analysis

**Fig. 1:**
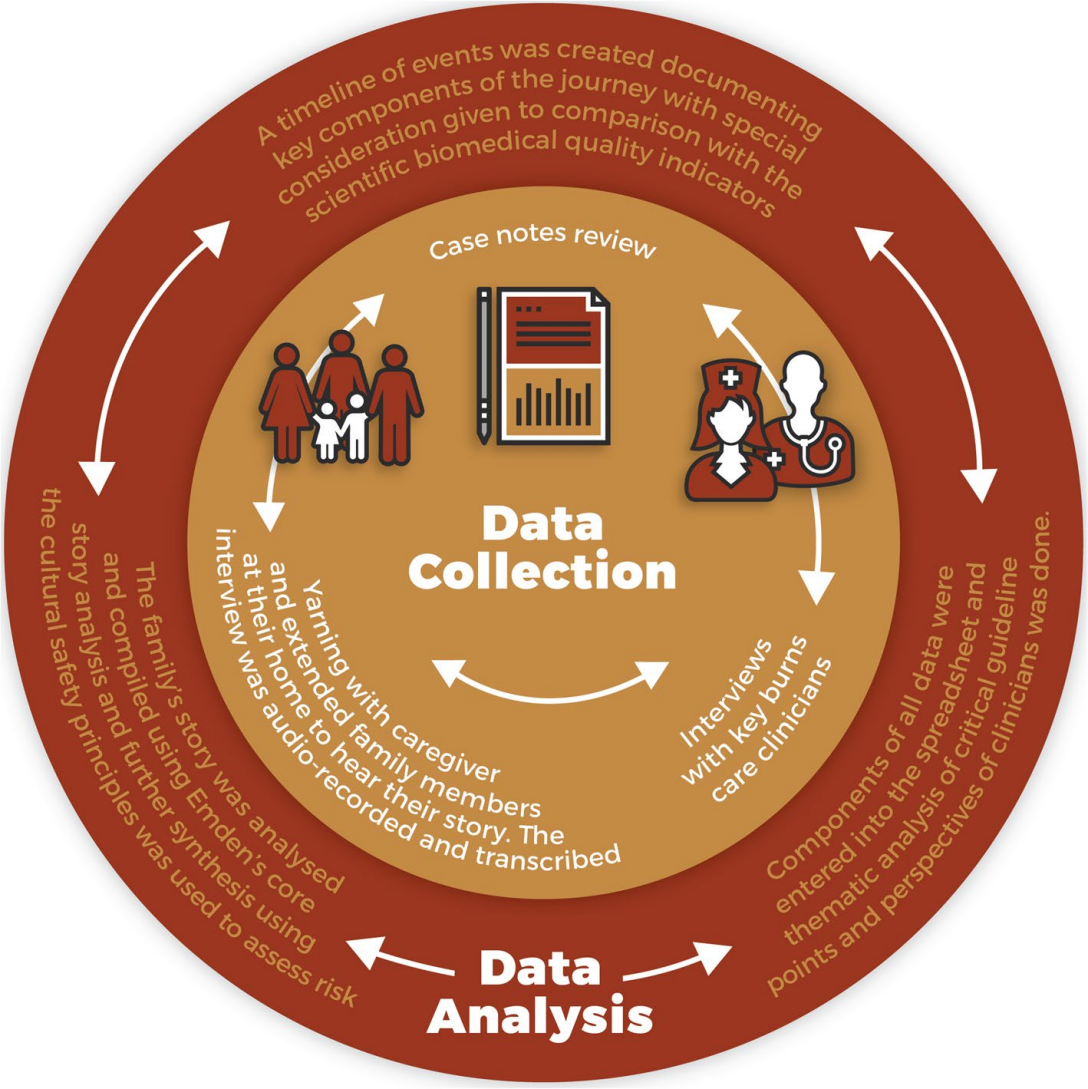
Data collection and data analysis mapping process

Data collection and analysis for the mapping process entailed four main stages, which were completed iteratively, but not sequentially (Fig. 1). These included yarning with the family, case note review, structured interviews with key burn care clinicians, and collaborative data analysis. In line with interface research [28, 29], Indigenous knowledges and understandings informed research yarning with authors TM and CR. Both of whom are Aboriginal woman and health researchers, linked closely through a cultural mentorship role to the main researcher (Author SF).

For the yarning with family, author SF was accompanied by author CR into the family’s home. Yarning [42] is an Indigenous cultural form of conversation and data gathering tool in research. This yarn was transcribed verbatim and analysis of this interview was through use of Emden’s core story analysis [26, 43] whereby a series of processes led to the creation of a de-identified core story that the participant confirmed for accuracy. This method of analysis provided a space to articulate the families’ journey holistically. These data were also analysed thematically using deductive methods [32] and input into the APJM tool spread sheets. Further synthesis of the yarning data provided the means to assess cultural safety and associated risk for the participating family. Consistent with the coming together of Indigenous and western knowledge, these processes contribute to reliability of data analysis and demonstrate our application of interface research.

The case note review and structured interviews with key burn care clinicians captured the quality components of the approaches to burn care, and the perspectives of healthcare providers. These data were also input into the APJM tool spreadsheets.

## Results

### Effectiveness of the modified APJM tool in identifying gaps in quality and cultural safety

#### Experience of the individual child and family

The APJM tool enabled the assessment of quality regarding the individual family’s experience of care in the healthcare system (Table 1). The family perceived that they experienced disrespectful care; they felt isolated and helpless during their child’s inpatient stay and vulnerable on discharge. The family also identified significant gaps relating to follow-up care and ease of access to rehabilitation. Synthesis of the yarning data (Supplementary Material 3) informed by the principles of cultural safety identified that care was most likely poorly experienced at the ‘sustained interface’ and ‘going home’ phase (Table 2). Such gaps in quality acknowledge the family’s experience of care as a result of fundamental differences in knowledge and understanding as reflected in the burn care health system.

**Table 1:** Results of PJM tool Spreadsheet Two. Indigenous concepts of health and healing and family and healthcare professional perspectives

Points in time—headings to elicit holistic views of health	Crisis	Getting help	Leaving competing obligations	Confronting the system	Sustained Interactions	Being away	Going home	Confronting competing needs
Caregiver's perspective	Accessible and appropriate care	Accessible and appropriate care	Care arranged for sibling and family contacted	Identification question asked Felt scared and ignored Social worker provided support	Communication was inconsistent Felt judged for not staying in the hospital Unable to work with subsequent extreme financial pressure and no access to disability pension Food vouchers infrequent and covered only very minimal amounts	Difficult to find care for sibling Increased burden on extended family for sibling care and visiting hospital Sibling difficult behaviour Financial support to cover part of fuel costs to drive to hospital each day. No PATS. Home bills left unpaid	Felt pushed out Discharged without confidence Psychological distress	Financial support to cover part of fuel costs to drive to hospital each day. No PATS Unable to return to work for almost one year Long appointments that meant whole day trips Sustained burden on extended family for sibling care
Child's perspective (6yo or >)	N/A as child < 6yo	N/A as child < 6yo	N/A as child < 6yo	N/A as child < 6yo	N/A as child < 6yo	N/A as child < 6yo	N/A as child < 6yo	N/A as child < 6yo
Referring Hospital/GP	N/A	Not able to speak to Ambulance worker Case Notes: identification question asked	Case Notes: consideration for care of sibling documented	N/A	N/A	N/A	N/A	N/A
AHW	No AHW employed	No AHW employed	No AHW employed	No AHW employed	No AHW employed	No AHW employed	No AHW employed	No AHW employed
A/ILO	N/A	N/A	N/A	Not notified child was Aboriginal whilst in ED	Supported family financially with fuel and food vouchers Helped with access to hospital child care for sibling	Not resourced to provide support care outside of the hospital or to those family outside of the hospital environment	Arranged by burn team	Not resourced to provide support
Ngangkari (Traditional Healer)	Not requested by caregiver. ? availability	Not requested by caregiver. ? availability	Not requested by caregiver. ? availability	Not requested by caregiver. ? availability	Not requested by caregiver. ? availability	Not requested by caregiver. ? availability	Not requested by caregiver. ? availability	Not requested by caregiver. ? availability
Burn Nurse	N/A	N/A	Notified via pager No support care provided	Attended ED on arrival of family Spent time with caregiver Provided caregiver with clothes to change in to	Time spent with caregiver M-F to ensure understanding Made caregiver feel comfortable with environment	Encouraged accessing extended family for support with sibling	Provided written instructions Gave some dressings	Attempts to make dual appointments
Occupational Therapist	N/A	N/A	N/A	Automatic referral received	Care provided in ICU	Attempts to make dual appointments	Discharge advice given	Attempts to make dual appointments
Physiotherapist	N/A	N/A	N/A	Automatic referral received	Positioning in ICU	Attempts to make dual appointments	Discharge advice given	Seen in scar clinic
Surgeon (medical staff)	N/A	N/A	N/A	Case Notes: Informed consent and surgical procedures	Case Notes: Informed consent and surgical procedures. Allowed caregiver to give consent over the phone for second and subsequent procedures	Case Notes: noted caregiver seen by social worker	Case Notes: Medical review prior to discharge	Case Notes: Wound and scar review as necessary
Psychologist	N/A	N/A	N/A	Case Notes: no input into care	Case Notes: no input into care	Case Notes: no input into care	Case Notes: no input into care	Case Notes: no input into care
Social Worker	N/A	N/A	N/A	Supported and sat with caregiver in ED Explained situation Explained presence of police officer and mandatory notifications	Supported caregiver with social health and welling Ensured access to fuel and food vouchers	Provide written evidence to support disability pension claim	Ensured access to fuel vouchers	Not resourced to provide support once discharged

**Table 2:** Yarning data synthesis of caregivers’ experience of culturally safe care and associated risk

Cultural safety Principle	Definition	In-Practice examples	Caregiver’s experience at holistic time points and associated level of risk				
			crisis	help	confront	sustain	home
Reflexivity	Reflect on practice, mutual respect	Respectful interactions	low	low	med	med	med
Dialogue	True engagement and consultation	Build rapport and dialogue with family alongside consideration of kinship arrangements and decision-making structures, particularly as they relate to children	low	low	med	med	med
Power imbalances	Minimise power differentials and maintain human dignity	Including Indigenous health workers in multidisciplinary teams	low	med	med	med	med
Decolonisation	Acknowledging the key role of colonising history in contemporary health outcomes for Aboriginal and Torres Strait Islander peoples	Ensuring equity in healthcare to achieve equity in health outcomes	low	med	med	high	high
Regardful care	Provide care that is regardful of culture and challenges the status quo of providing care that is regardless of culture	Patient-centred care; where the context for the child and their family drives care decisions	low	low	high	high	high

#### Perspective of health care providers

Mapping data identified that healthcare providers were neither resourced nor supported to provide best care following patient discharge, and there were limited options for referral to community based social workers (Table 1). APJM also showed healthcare providers mostly conform to the evidence underpinning medical aspects of burn care. There was no Aboriginal Health Worker AHW employed at the site, nor was any input from a psychologist involved in the care (Table 3).

**Table 3:** Results PJM tool Spreadsheet One. Scientific standards and family and healthcare providers meeting standards

**Burn care standards **[30–32, 34, 35]	**The injury**	**Emergency care**	**Ambulatory care**	**Admission**	**In-patient care**	**Discharge**	**Rehabilitation**
Standards achieved by healthcare service and healthcare professionals	⋅ 20 min cool running water within first 3 h ⋅ Remove jewellery and clothing ⋅ Cover with non-adherent dressing ⋅ Seek medical assistance ⋅ Keep warm ⋅ Provide access to basic online first aid training on burn injury to target the community ⋅ Ensure first aid courses contain burn first aid content		⋅ Burns greater than 5% in children ⋅ Full Thickness burns greater than 5% ⋅ Burns of special areas ⋅ Burns in very young ⋅ Children up to their 16th birthday should be transferred to a children's burn unit ⋅ Metro clients access tertiary facilities directly, and outer regions require routine links to tertiary facilities ⋅ Access to specialist service	⋅ Consult with a burn surgeon ⋅ Access to physiotherapy, · occupational therapy, social work, speech pathology, nutritional support, clinical psychology ⋅ Ambulatory burn clinic provides assessment and dressing of minor and non-severe burns, rehabilitation interventions, follow-up burn dressing and skin graft management for patients after discharge ⋅ long-term scar management and symptom control ⋅ patient and family teaching and support ⋅ ongoing complication risk management and treatment ⋅ advisory service to other hospitals, healthcare professionals and community	⋅ Social worker undertakes thorough psychosocial assessment to review family history and address psychosocial issues in the acute phase ⋅ Accurate assessment undertaken in the ED in accordance with the admission guidelines for individual burn unit ⋅ Laser Doppler Imaging to assess depth ⋅ Rehabilitation starts on admission and whole patient and family are considered when addressing rehabilitation needs ⋅ Care plan is developed and documented and reviewed on a continual basis . Case management is commenced on admission ⋅ Allied health contributes to all stages of continuum of care guided by clinical practice guidelines ⋅ Nurses provide holistic care and are integral to patient care from point of admission to rehabilitation to ambulatory care ⋅ Multi-disciplinary teams coordinate individual clinical pathways ⋅ Each discipline contributes to treatment plan	⋅ Social work and clinical psychology provide assessment and intervention ⋅ Dietician assessment for burns > 10%, < 1yo, burn to mouth/hands ⋅ Nursing staff work closely with comprehensive pain management service incorporating a range of modalities and including non-pharmacological and complementary therapies ⋅ Care plan incorporates rehabilitation throughout all stages of care starting at time of injury and family are considered when addressing rehabilitation needs ⋅ Major burn patients should be assessed within 24 h of admission by physiotherapy OR occupational therapy ⋅ Multidisciplinary plan of care · Allied health contributes to all stages of continuum of care guided by clinical practice guidelines ⋅ Multi-disciplinary teams coordinate individual clinical pathways ⋅ Receive multi-disciplinary inpatient care ⋅ Each discipline contributes to treatment plan ⋅ Burn injury team liaises with microbiology and infection control ⋅ The burn injury team works closely with the pharmacist in the management of care ⋅ State-wide e-health service supporting consultant-led on-call advisory service ⋅ Patients managed in ICU require coordination of wound care by burn care nurses ⋅ Access to pathology services ⋅ Nursing staff provide holistic care ⋅ 24 h access to operation rooms ⋅ Paediatric treatment rooms · Child protection unit involvement	⋅ Pharmacist to provide regular information to child, family, carer on medication at admission and discharge ⋅ Allied health contributes to all stages of continuum of care guided by clinical practice guidelines ⋅ Social work and clinical psychology provide assessment and intervention ⋅ Address psychosocial issues, prior to discharge . Case management for complex cases continues throughout long-term care to facilitate periodic re-assessment and monitor changes in functionality ⋅ Patients to receive 'Nutrition for burns' pamphlet prior to discharge
Standards not achieved by healthcare service and healthcare professionals			⋅ Provide 7 day/week ambulatory burn service co-located with acute inpatient burn unit ⋅ Burn injury patients have access to ‘hospital-in-the-home’ services post inpatient discharge	⋅ Clinical psychology provides assessment and intervention at admission	⋅ Comprehensive nursing care plan developed in consultation with patient and/or caregiver on admission to unit	⋅ Facilitated early discharge by accessing ‘hospital-in-the-home’ services, and by using a step down to local non-tertiary hospital for transition to rehabilitation	⋅ Use telehealth for ongoing post-acute care of burn patients ⋅ Rehabilitation team provides referral to external rehabilitation facilities for ongoing management ⋅ Be referred to OT/physio at local services where available, with support from burn unit therapists ⋅ Patients and families continue to receive psychosocial intervention and refer to other agencies where required
Standards not applicable for this burn care journey		⋅ Inhalation, electrical, circumferential and chemical burns ⋅ Burns with illness ⋅ Burns with major trauma ⋅ Any burn where the referring worker requires management or advice from the paediatric burn service ⋅ Burn injury with suspicion of non-accidental injury ⋅ Appropriate communication and management instigated for interstate transfers within 4 h ⋅ The facility who has first contact with the burn injury contacts the unit for support and advice ⋅ For minor burns, communication with unit regardless of confidence in assessment and plan of care ⋅ For moderate burn, communicate with unit early and adopt recommended guidelines ⋅ Laser Doppler technology is used to assess depth ⋅ Initial assessment in ED where staff communicate with state unit, providing 24-h turnaround service via email images for clinical advice	· accept patients referred from a hospital emergency department, general practitioners, other hospitals, community health services, or self-referred ⋅ burn injury of up to 10% of total body surface area may be managed on an ambulatory basis · Outpatient community care may include home, school, pre-school and workplace visits ⋅ Referral to dietician if deemed to be at nutritional risk; followed by nutritional assessment for social and cultural needs ⋅ Use of step-down facility to allow access to ambulatory care services for rural and remote families ⋅ patients with a burn who require surgery, with interim burn care until the day of surgery	⋅ Emergency surgery within 24 h post-deep circumferential burn ⋅ Access to Burn Unit is dependent on post-assessment classification of the burn injury using E-health Outreach Service via non-specialist centres for regional/rural/remote	⋅ Education teacher on daily basis ⋅ Psychosocial assessment focussing on the accident causing injury and family member’s perceptions around this, past experiences of trauma, family dynamics, cultural and socio-economic factors, barriers to coping and family strengths and supports ⋅ Long term access to psychological support	⋅ Provide access to sub/acute/step-down facilities ⋅ Referral to community agencies for support at home if required	
Standards unable to be assessed			⋅ Staff attending burn patients in outpatient setting observe standard precautions at all times, including hand hygiene and aseptic non-touch technique and relevant PPE				⋅ Step-down facilities are linked to acute services to achieve a seamless continuum of care ⋅ Provide access to burn camps for children ⋅ Contribute to cooperation between family and school ⋅ Visit school with burn team to educate
Data from Case Notes and discussions (where able) regarding how standards were/were not applied							
Caregiver	Had completed first aid training		Accessed emergency ambulance care	Travelled in private car to appointments. From daily dressing to once every 6 weeks	Time in emergency department then transferred to ICU	Four days in ICU (and staying at home at nights) and four weeks in surgical unit (staying at home and sometimes in hospital)	Travelled home in private car. Felt hurried out and inadequately prepared to provide necessary at-home care
Family	N/A		Contacted by phone after accident occurred	Travelled in private care with caregiver occasionally	Arrived at hospital after admission to ICU	Visited often in private car	
Aboriginal Health Worker (AHW)	No AHW employed		No AHW employed	No AHW employed	No AHW employed	No AHW employed	No AHW employed
ACCHS	Not accessed by the family		Not utilised by the family	Not accessed by the family	Not utilised by the family	Not utilised by the family	Not utilised by the family
Emergency Care Provider	Not able to contact place of injury or those present at time of injury		Not able to contact Ambulance worker Case Notes: Mandatory notifications made	N/A	N/A	N/A	N/A
Surgeon	N/A		N/A	Consults as necessary	Surgical assessment within 4 h of admission to hospital	Surgical intervention	Discharge note made
Burn Nurse	N/A		N/A	Arranged care appointments and supported caregiver in minimising time spent in hospital	Support transition to ICU and then to ward. In regular contact with caregiver and giving constant information	Developed initial care plan. Led case conferences with medical staff. Involved multidisciplinary team. Reviewed at least daily	Gave information regarding required care Arranged follow-up appointments
A/ILO	N/A			No support provision	Not notified	On A/ILO list. Seen and offered support. Did not attend case conferences	Seen prior to discharge and support offered
Traditional Healer	N/A		No traditional healer employed	No traditional healer employed	No traditional healer employed	No traditional healer employed	No traditional healer employed
Occupational Therapist	N/A		N/A	Consults in scar clinic	Assessed within 8 h of admission	In patient care provided. Attended case conference Input into care plan	Discharge note made
Physiotherapist	N/A		N/A	Consults in scar clinic	Assessed within 24 h of admission	In patient care provided. Attended case conference Input into care plan	Discharge note made
Psychologist	N/A		No input into care. Not able to be contacted	No input into care. Not able to be contacted	No input into care. Not able to be contacted	No input into care. Not able to be contacted	No input into care. Not able to be contacted
Social Worker	N/A		Attended ED. Supported, engaged and explained	No input into care	Able to provide support to caregiver and available for all level 1 trauma	Provided initial assessment of caregiver, supported, engaged and provided intervention where necessary and supported access to fuel and food vouchers. Attended case conference	Discharge note made

#### Assessment of the health system

The APJM tool was able to identify where there are gaps in the healthcare system regarding quality of burn care for Aboriginal and Torres Strait Islander children and families. Burn care was mostly delivered in line with the prescribed indicators of biomedical quality; e.g. first aid was given, burn specialist assessment was within the stipulated time-frame and multi-disciplinary care was provided. However, contribution by the Aboriginal/Indigenous liaison officer (A/ILO) was both late in the timeline of clinical care, and limited. Access to rehabilitation services outside of the tertiary healthcare environment was reduced and did not meet standards (Table 3).

## Discussion

### Implications of the APJM tool

The innovation in using the knowledge interface and Indigenous research methods, combined with the principles of cultural safety and guided specifically by the principle of reflexivity [44] as it relates to researchers and health care with Aboriginal and Torres Strait Islander people, enabled the implications of developing and applying the APJM to be considered. Engagement in reflexive practice provided a space to inform this process from a culturally safe and holistic health standpoint.

Through application of the APJM tool, we have shown feasibility of its use in the burn care journeys of Aboriginal and Torres Strait Islander families such that it enables an exploration of the multiple layers of experience in the health care system. This includes the system, service and individual with a holistic model. With the engagement of Indigenous knowledge, we have been able to capture gaps in quality that relate to more than biomedical quality evidence. This APJM research has shown that while complex disparities being experienced by Aboriginal and Torres Strait Islander children and families, quality is able to be explored thoroughly when using tools that address all aspects of quality. With these aspects considered, APJM is time intensive and challenging to engage clinicians.

Application of the APJM tool was undertaken over four months (approximately 120 h) in 2018. The tool was lengthy and could be reduced by the inclusion of only those standards specific to the jurisdiction where the tool is being used. Testing of the tool with regional/remote families and with older children is required. Recruitment and engagement of family members in mapping was enhanced by existing relationships with the mother. Having the grandparents present at the family interview contributed to a deeper understanding. The family interview was facilitated with an Aboriginal woman as a co-researcher, supporting ethical research. The interview was conducted in the family home, on the basis the family were more likely to feel secure in their own space, and dinner was supplied though study funds.

### APJM for use in complex quality investigations

Results from this study show existing APJM tools [26] are able to be modified to investigate quality and cultural safety in burn care for Aboriginal and Torres Strait Islander children and families in a tertiary setting. Through application of the tool, new levels of understandings and recommendations for changes in practice which can alter the experience of those receiving the care were able to be identified. The implications of engaging an innovative approach through interface research show quality improvement is able to be considered from more than regulation or performance perspective. An exploration of complex disparities was enabled that incorporated patient experiences at the individual, service and system level. This study was limited due to being only one case with no control, and a similar study with a non-Indigenous family may have generated similar issues.

These findings confirm that retrospective data is useful in assessing healthcare quality in patient journeys, as well as interactions between various components of quality in this setting. The APJM tool enabled assessment of performance, regulatory constraints and patient experience in tandem. Analysis of these data, using Emden’s analysis method [43, 45] and thematic analysis [32] gave insight into the families’ journey of quality in burn care. The tool also gave burn healthcare providers the opportunity to externalise and reflect on their capabilities and the care they provide. As a result, use of APJM provided a space for healthcare providers to consider how to improve and innovate within their own practice through reflexivity. While existing professional relationships with the lead burns nurse made access to relevant clinicians more successful due to this nurse’s influence on facilitating clinician availability, it was sometimes difficult to engage busy clinicians with the mapping process when seeking to clarify and understand key points and interactions within the journey. This reflects the findings of the Managing Two World Together Project where clinician engagement was promoted by collaborative involvement in the research and development of tools, as compared with externally-developed tools imposed upon them [26]. Therefore provision of more information on the processes and aims of APJM may enhance participation by busy clinicians.

This preliminary analysis did not include responding to the findings to improve communication, reduce perceived differential treatment or enhance access to post-discharge care. Further considerations need to be made to determine how best to work with multidisciplinary burns teams and healthcare services to effectively plan and implement improvements in burn care for Aboriginal and Torres Strait Islander children and families. Existing QI frameworks and engagement in reflexivity by healthcare practitioners may be key aspects of future approaches, however there does exist opportunities for immediate QI considerations. This includes the employment of Aboriginal Liaison Officers as part of burn teams, appropriate resource allocations and increased focus on Aboriginal and Torres Strait Islander aspects to burn care in team meetings (clinical and research).

## Conclusions

The APJM tool aims to facilitate the exploration of complex patient journeys following a burn injury, the increase in knowledge of what works well and what needs improvement in the healthcare system for Aboriginal and Torres Strait Islander children and families. Whilst many methods and methodological approaches to improve the quality and safety of healthcare exist, very few address the knowledge interface of Indigenous and western health knowledges, nor provide opportunity for children to have a voice. In undertaking this study, we have developed a tool enabling research of burn care quality at the knowledge interface, with explicit consideration of more holistic, fluid and culturally safe models of healthcare.

## Supplementary Information

**Additional file 1.** 

**Additional file 2.** 

**Additional file 3.**

## Data Availability

The datasets generated during and/or analysed during the study are not publicly available due to ethical restrictions but may be available from the corresponding author on reasonable request.

## Abbreviations

ILO: Indigenous Liaison Officer
ALO: Aboriginal Liaison Officer
APJM: Aboriginal Patient Journey Mapping
QI: Quality Improvement
