# Supplementary material for: Patient journey mapping to investigate quality and cultural safety in burn care for Aboriginal and Torres Strait Islander children and families – development, application and implications
Source: BMC Health Serv Res. 2022 Nov 28;22:1428. doi: 10.1186/s12913-022-08754-0 (PMC9703784; doi:10.1186/s12913-022-08754-0)
Supplement: Supplementary file 3 — Additional file 3. [file 12913_2022_8754_MOESM3_ESM.docx]

**Supplementary Material 1. Emden’s core story analysis of family narrative.**

A mother, Kate, her toddler son, Tom and baby Ava attended a planned appointment concerning Tom. During the appointment, Ava became hungry and restless and Kate organised for her bottle to be heated. Ava suffered an unintentional serious scald injury. Kate’s instinct was to pull Ava from the child’s restraint in which she was laying. Instructed by staff to take Ava to the shower for first aid, Kate removed Ava’s clothes. As Kate removed Ava’s clothes, she saw the ‘blistering and her skin just going down the drain’. She felt like *‘the worst person in the whole world’* and was unsure of what to do.

On-site staff had phoned triple zero for emergency care and were arranging for the care of Tom with family contacts from Kate’s phone. Kate was hysterical and staff removed other people in close vicinity from the area. When the ambulance arrived, paramedics wrapped Ava in glad-wrap and they asked Kate if she was going to come to the hospital with them. They also asked if Ava was Aboriginal and/or Torres Strait Islander. Kate said yes to both. There was a police officer in attendance in the Ambulance en-route to the tertiary children’s hospital.

On arrival at the hospital, Ava was taken into the resuscitation room. The emergency response team was in attendance and ‘it was so crazy in that room’, Kate said. Emergency team staff said to Kate: ‘we’re going to put you over in this corner so you can stay with her but you can’t be in the way’. Kate was asked Ava’s weight and she said she ‘couldn’t even hear her [Ava]. There wasn’t crying, wasn’t anything, [she] didn’t even know at that stage if she [Ava] was even alive, like, [she] couldn’t see her, couldn’t hear her’. ‘I wanted to know what was going on but I couldn’t get those answers and then the only people at that stage that were talking to me was a police officer and a social worker’. The lead emergency doctor introduced themselves to Kate and said Ava was in good hands. The social worker, police officer and doctors kept asking Kate questions. ‘Everyone [was] just asking me [Kate] all these questions and I [Kate] had to keep saying the same thing over again, again’. Kate was asked once by staff if Ava was Aboriginal and/or Torres Strait Islander. The social worker was a great support to Kate, was the best source of information, and made Kate feel as comfortable as possible.

Once Ava was stable, she was transferred to the PICU and Kate, who was still in wet clothes from the shower, was given warm blankets. Kate’s parents arrived at the hospital and Kate retold the story to them. While Ava was receiving treatment in PICU, Kate and her family sat outside ‘in the family room and just [were] waiting and waiting and waiting’. After what seemed like an eternity to Kate and her family, a PICU doctor finally came out and said that Ava was stable. The doctor also assessed Kate’s burns, which were superficial and did not require any medical intervention. Kate was provided access to a shower and some scrubs to change into. She was desperate to talk to her husband, Ava’s father, who was uncontactable. Kate and her parents felt left out and did not know what was happening with Ava during the first few hours of treatment. Kate’s mother said ‘…to be left in the dark like that – would have been nice if we had someone come in to say, look, there’s been a few problems, you know, but you don’t know’.

After some time, another doctor came out to give an update on Ava’s condition. This time Kate was invited into the PICU to be with Ava but was told: ‘you won’t be able to see her again for the rest of the day so, sort of, appreciate – appreciate it’. Kate felt like they were taking Ava away and that she wouldn’t be allowed to see her again, ever. Kate’s mother went in with her for support. They weren’t allowed to touch Ava and had to wear gloves, a mask and a gown.

It was the hardest thing for Kate to hear that she was not allowed to touch her baby in the time of most need. Kate’s mother was in shock at the sight of Ava. The heat in Ava’s room was unbearable.

After visiting Ava for a short time, Kate’s parents took Kate home to see her son, Tom. On arriving at her husband’s parents’ home, who had collected Tom from the place of injury, Kate held Tom and cried. Kate was very surprised to learn from her in-laws that ‘all while we were at the hospital, low and behold to me, ah, there was an investigation happening’. She had thought it was strange when the police officer at the hospital had told her that ‘it’s okay. It’s been classed as an accident. I’m going’. Kate was so worried and felt she had been left in the dark. This was an assessment regarding child abuse and she ‘feared they were going to take Ava and Tom away’. Kate’s in-laws told Kate her car was unable to be taken from the place of injury due to the police investigation. The car was released a few hours after the incident. Kate never collected the clothes Ava was wearing on that day.

Ava stayed in the PICU for five nights. Day two was hard for Kate and her parents. This was when the severity of Ava’s burns became overtly evident. ‘Overhearing staff talk about the severity [of Ava’s condition] was the worst’. Kate’s mother and father supported Kate during those early days and provided care for Tom. The family travelled at least one hour to and from the hospital each day as there was only one PICU parent’s room and that was occupied. Kate arranged for weekly car parking to reduce the cost of casual parking.

During Ava’s stay in the PICU, communication regarding Ava’s condition and care from staff got better and better. Kate felt continuity of staff was very helpful and in general, ‘the nurses explained a lot’. Sometimes however, inconsistency of messages made Ava’s time in PICU somewhat confusing for Kate and her family. Nonetheless, the staff also made sure Ava was safe and that only family who were allowed to see her, came in. After Kate and her family left at night to go home, Kate would phone to see how Ava was. ‘The PICU nursing staff would tell me everything over the phone. I would say that I want every update that’s happened since I left. I want to know if there are any doctors’ notes and I want everything. It was really good. We felt very comfortable and it helped me go to sleep’.

Ava was moved from the PICU to the surgical ward in the hospital on day six of her admission. The main burn nurse, Lucy organised for Kate and her family to be introduced to the surgical ward nurses prior to moving. Kate was asked by a staff member of the surgical ward if Ava was Aboriginal and/or Torres Strait Islander. Lucy met with Kate frequently, especially in the early days of Ava’s admission and supported Kate in holding Ava for the first time since the injury. Kate said, ‘I literally cried, I was so happy to be able to finally hold her’.

Tom would visit Ava daily with Kate unless it was a day-care day. However, it was not until Ava had been in hospital for a few days that Kate became aware that there was a child’s crèche available. This would have been helpful to know earlier as Tom was not allowed in the PICU for very long at any given time. Over the next four weeks Kate started using the crèche for Tom, however Tom became very clingy and needed to sleep in Kate’s bed at night. He also became angry and would throw a lot of tantrums. Family support was very important in terms of helping with Tom, especially as he became increasingly challenging. Tom wanted Kate and ‘when I [Kate] was there, I [she] wasn’t really emotionally there’.

Over the following weeks, Kate arranged her life around being with Ava in the hospital. She stayed at home at night during the weekdays, and overnight in the hospital with Ava on the weekends. Kate also drove several hours once a week to visit her husband. Tom would mostly accompany Kate on these visits, however he stayed with Kate’s parents as necessary. Kate described every day to be like ‘ground-hog day’.

Kate experienced extreme financial stress for the period of Ava’s time in hospital. Kate ‘had no idea how I would get through’. She would sacrifice bills and would pay only what was most necessary and leave what she could not pay. At the time of the injury, Kate had been planning on returning to work to support herself and her two children. This never happened. Meals at the hospital were expensive, and there was very little suitable food for children. Sometimes Kate’s parents would pay for the meals, which would cost hundreds of dollars if they were there seven times in a week. Kate would also try and pack her lunch to reduce costs. The social worker organised fuel vouchers. This was almost $200 over the four weeks. Kate was also supported with 14 meal vouchers. Kate’s application for the carer’s pension with Centrelink was declined as she was deemed not eligible because Ava was too young and it was expected that she already be in 24-hour care.

Ava had multiple surgeries that Kate was required to give consent for. Kate said that ‘for her first lot of skin grafts they actually sat in there and answered every single question we had, no matter how stupid it was’. Many of the times following the initial consent, medical staff would seek verbal consent over the phone for the surgical procedures given Kate lived so far away and could not be there in person all of the time. Kate would go to the hospital after the surgery and be there when Ava came out. There was nothing Kate felt she didn’t understand in the consenting process and the process got quicker for each surgery that Ava had. *‘I’*m putting my absolute trust in you that, like, yes, I want to know what you’re doing but at the same time I’m not going to go sit there looking for a better solution because that’s going to delay her care’.

On one occasion, there was confusion and the nurse was rude to Kate over the phone. ‘…I’ve treated them with the utmost respect and courtesy, everything, and then just if they – being spoken to like that on the phone it was, like, that’s not on…I deserve some respect’. Kate reported this to the lead burn nurse, Lucy the next day. There was also one night when nursing staff would not give information about Ava over the phone, even though it had been given every other night. Sometimes Kate felt it was difficult to get messages left for staff on the next shift, especially about expected visitors. This was the result of a communication breakdown between staff. Again, Kate reported these things to Lucy who rectified the communication problems. Mostly though, Kate was happy with the communication and care from staff.

Not only did Kate feel consistently guilty about Ava’s injury, she was often made to feel guilty about not coming in to be with Ava. However, when she did come in, she was told off when Tom or her nieces and nephews were being too loud. She also often felt judged by healthcare professionals in the cafeteria when her extended family were visiting. ‘You could tell from the vibe from some of the other doctors and stuff when you’re in the cafeteria, like, you’re being loud and you’re this and you’re that and, like, I’m trying, you know’.

For a long time, the nurses provided all of the care for Ava, and Kate thought it was mostly nice to have their support. However, when Ava moved to care that was not one on one, Kate was expected to do everything for Ava, without having been taught how. Kate said that ‘no-one actually sat there and taught us how, they just thought we knew’. Kate was disappointed by this.

After four long weeks, Kate was ecstatic to be leaving the hospital and going home with Ava. She was told to take everything from the room to care for the wounds as it would only be thrown out. Kate was given very quick instructions on how to change Ava’s dressings on the morning she left the hospital. She left the hospital feeling immensely apprehensive about having Ava at home and having to do the wound care all by herself. This was made better by the communication with hospital clinical staff upon being home. This was especially true regarding the care of Ava’s dressings. Kate was very disappointed to learn in the discharge summary upon being home that Ava had been resuscitated twice in the PICU and she had not been told.

Outpatient appointments at the hospital after discharge meant getting up very early, with Tom staying at Kate’s parents’ house the night before so as not to have to wake him at 6:00am. The appointments were almost three hours long with one hour’s drive each side of the appointment. They started as twice a week, then moved to once per week, then fortnightly, then every three weeks, every four weeks and then every six weeks. Ava would get very upset and restless during the long outpatient appointments.

Access to Close the Gap helped Kate with the cost of medications and bandages post-discharge. Kate was not sure she would have been able to afford all of these extra products if it had not been for this scheme. Kate continues to worry about the rising costs of care for Ava over her long-term care journey. Kate has also had to purchase new things to keep Ava protected from the sun, such as special swimwear. Kate’s parents keep their own stock of cream to care for Ava at their home. The creams and wash are expensive, and all of the family have struggled with these costs. With Kate’s return to work, the financial stress has decreased somewhat. The cost of Ava’s special garments is extraordinary, and Kate was thankful she did not need to pay for these. She would not have been able to afford them.

While Kate was thrilled to be home, the nights were difficult, especially when Ava’s wounds were itchy. It was sometimes very difficult for her to get to sleep. For Kate, taking the silicone off herself was difficult because it made her think she was ripping the skin off. This reminded her of the time of the injury. Although Kate had the support of the social worker while in hospital, this worker’s obligations stopped as soon as we left the hospital. Kate was offered a psychologist once Ava was discharged from the hospital to work with. Kate continues to be concerned about Ava showing signs of post-traumatic stress disorder.

Ava continues to get better. For the moment, outpatient appointments are six-weekly, and Ava has started laser treatment for her scars. Kate continues to successfully manage Ava’s care with Tom, work and other commitments.
